# Glutamine synthesis pathway promotes the survival and recurrence of dormant tumor cells in prostate cancer

**DOI:** 10.1038/s41420-026-03282-w

**Published:** 2026-08-27

**Authors:** Bing Zhao, Qingwei Meng, Jun Zhou, Kun He, Hekang Ding, Jing Wang, Hailiang Hu, Lingfan Xu

**Affiliations:** 1 Department of Geriatrics, Anhui Key Laboratory of Geriatric Immunology and Nutritional Therapy, the First Affiliated Hospital of USTC, Division of Life Sciences and Medicine, University of Science and Technology of China, Hefei, 230001 China; 2 Department of Urology, the First Affiliated Hospital of Anhui Medical University, Hefei, 230001 China; 3 Institute of Urology, Anhui Medical University, Hefei, 230001 China; 4 Anhui Province Key Laboratory of Genitourinary Diseases, Anhui Medical University, Hefei, 230001 China; 5 Department of Urologic Oncology, the First Affiliated Hospital of USTC, Division of Life Sciences and Medicine, University of Science and Technology of China, Hefei, 230031 China; 6 Department of Biochemistry, SUSTech Homeostatic Medicine Institute, School of Medicine, Southern University of Science and Technology, Shenzhen, 518055 China; 7 Key University Laboratory of Metabolism and Health of Guangdong, Southern University of Science and Technology, Shenzhen, 518055 China

**Keywords:** Prostate cancer, Prostate cancer

## Abstract

Cancer cell dormancy presents a major obstacle to curative therapy, particularly in metastatic prostate cancer. Although the initial efficacy of hormonal therapy is satisfying, tumors will inevitably develop resistance. Dormant tumor cells may act as the initial source for the tumor recurrence, but the underlying mechanism remains largely unknown. Here we report that the glutamine synthesis pathway is required for cellular dormancy under androgen-deprived conditions. The key enzyme, glutamine synthetase (encoded by glutamate-ammonia ligase, GLUL), is upregulated during hormonal therapy, which leads to overabundant glutamine as a nutrient and an energy storage for tumor recurrence. The negative modulation of androgen receptor (AR) on GLUL enables activation of glutamine anabolism when AR is inhibited. Pharmacologically inhibiting GS’s activity along with hormonal therapy results in cell apoptosis instead of dormancy, which significantly delays the progression of tumors into advanced PCa. In summary, we provide a novel perspective of how hormonal therapy eventually fails and highlight a combination treatment strategy to overcome therapeutic resistance of current hormonal therapy.

## Introduction

Prostate cancer (PCa) is a worldwide malignancy in male with morbidity of around 1.5 million new cases annually. Of note, approximately 10% of newly-diagnosed patients present with metastasis, particularly at bone sites, that can not be cured by surgery or radiation therapy [1, 2]. Hormonal therapy, including androgen deprivation therapy (ADT) and next generations of androgen receptor (AR)-targeted therapy, is the mainstay management for prostate cancer since most primary cases are androgen-dependent. In spite of initial efficacy, almost every patient will eventually develop resistance and progress into the castration-resistant stage where effective management is rather limited. Understanding the mechanistic failure of hormonal therapy remains challenging in clinical practice [3].

Cellular dormancy is an important self-protective mechanism for cancer cells under environmental stress, such as various cancer-associated treatments [4]. Tumor dormant cells are non-cycling but can be reactivated when treatment is terminated or cells have been adapted to the harsh circumstance. Such cells can usually be detected in metastatic lesions, but still lie dormant in orthotopic organs [5–7]. A good example is the hormone-dependent cancer (e.g., PCa or breast cancer), which is prone to have bone metastasis [8–10]. In PCa, it is very common to observe distant metastasis or in situ recurrence of tumor after years of remission for patients who have achieved a clinical “complete response”. One of the possible explanations is that both the bone niche and prostate environment produce pro-dormancy factors (e.g., CXCL12) that enable niche engagement and maintenance of dormancy [11]. However, the underlying mechanism of how dormancy prevents tumor cells from apoptosis and helps tumor cells escape hormonal therapy remains largely unknown.

Metabolic reprogramming is a hallmark for cancer initiation and development [12–14]. We have previously revealed that glutamine metabolism is significantly altered along with drug resistance and tumor development [15–17]. One of the key metabolic features is the isoform switch of glutaminase (GLS1) driving PCa into advanced stages where tumor cells are addicted to glutamine, not to androgen anymore [15]. However, the question how glutamine utilization is halted during hormonal therapy but highly active in castration-resistant PCa (CRPC) has not been answered yet. In the current study, we further dissect the connection between glutamine metabolism and cellular dormancy and how this “on-off” transition contributes to therapeutic resistance and disease progression. We report here that hormonal therapy leads PCa cells to a dormant status through enhanced glutamine synthesis activity. It is achieved by the upregulation of the rate-limiting enzyme, glutamine synthetase (GS), which is negatively modulated by the AR signaling. AR-directed therapy triggers the self-protective mechanism for dormant cells, accompanied by accumulating intracellular glutamine, which is the critical source that advanced tumor cells rely on. Therefore, inhibiting glutamine synthesis pathway on one hand enhances the efficacy of hormonal therapy by causing increased cell death, on the other hand delays tumor recurrence by cutting off the main energy-providing source. This strategy is therefore promising to diminish the mortality of CRPC and improve the outcome of metastatic PCa patients.

## Results

### Hormonal therapy induces glutamine accumulation in prostate cancer

**Fig. 1:**
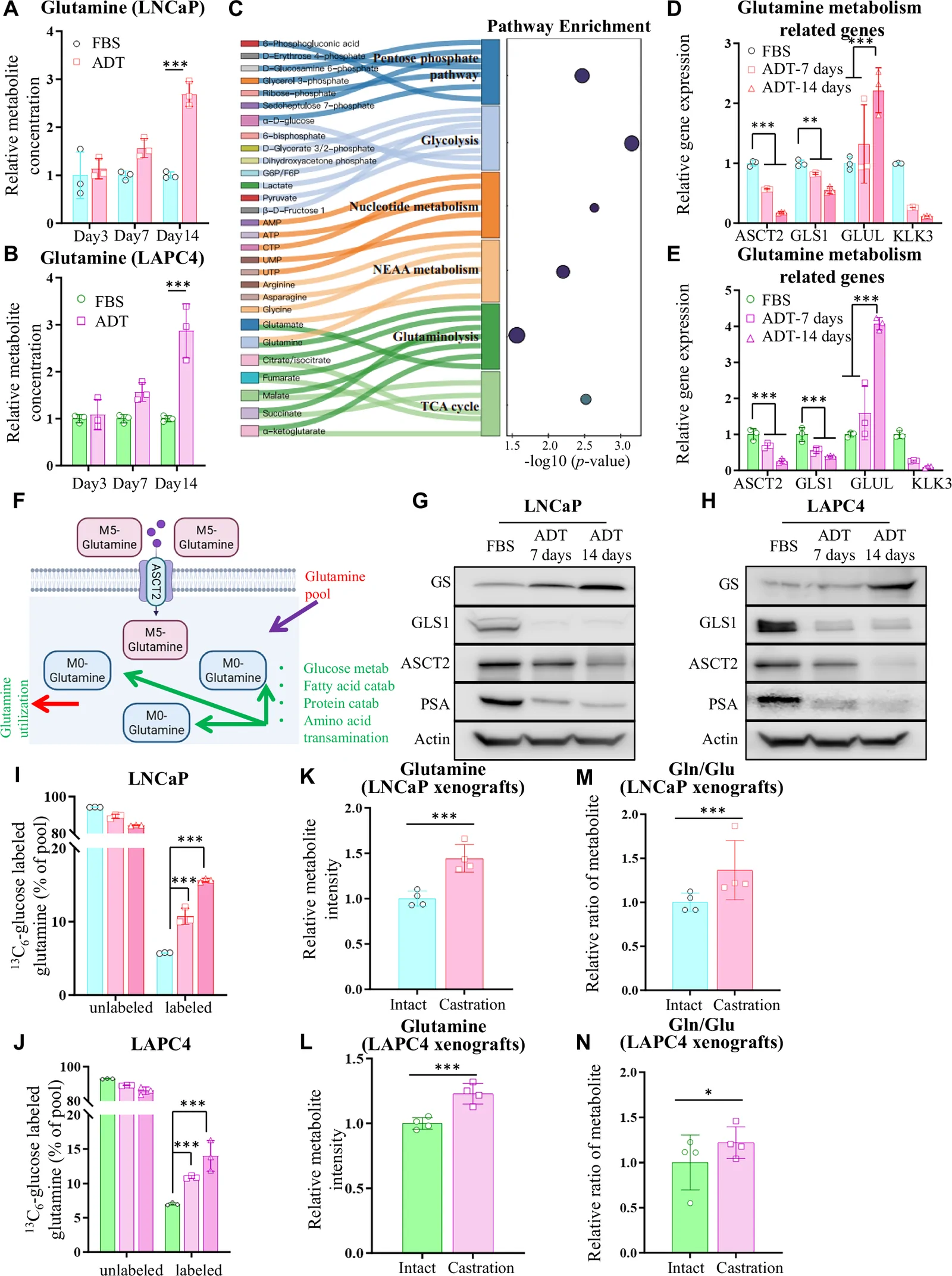
Hormonal therapy induces glutamine accumulation in prostate cancer. **A**, **B** Intracellular glutamine levels measured in LNCaP or LAPC4 cells treated with either FBS or ADT at indicated time points. **C** Pathway Enrichment analysis of decreased metabolites after ADT for 14 days, analyzed by MetaboAnalyst 5.0 based on the KEGG database. **D**, **E** Transcript levels of ASCT2, GLS1, GLUL, and KLK3 in LNCaP and LAPC4 cells treated by ADT for 7 and 14 days. KLK3 served as a positive control. **F** Schematic overview of the composition of intracellular glutamine pool. Basically, there are three pathways for glutamine metabolism: glutamine uptake, glutamine utilization (red arrow), and glutamine synthesis (green arrow). **G**, **H** The expressions of glutamine synthetase (GS), glutaminase (GLS1), and ASCT2 in LNCaP and LAPC4 cells treated with or without androgen deprivation. PSA served as a positive control. **I**, **J** ^13^C_6_-glucose tracing analysis showing the incorporation of glucose into glutamine in LNCaP and LAPC4 cells treated with ADT for 7 and 14 days. **K**, **L** Liquid chromatography mass spectrometry (LC-MS) showing relative glutamine intensity in LNCaP and LAPC4 xenografts after castration. **M**, **N** The ratio of Gln/ Glu calculated in LNCaP and LAPC4 xenografts. Data are depicted as mean ± s.d. *P < 0.05, **P < 0.01 and ***P < 0.001.

The progression from primary PCa towards to advanced PCa (e.g., CRPC) induced by hormonal therapy results in significant metabolic reprogramming. One of the hallmarks is glutamine addiction [15, 16]. To investigate how glutamine metabolism changes during ADT, we employed metabolite kits to measure glutamine and glutamate levels in LNCaP and LAPC4 cells, two androgen-dependent PCa cell models that represent primary PCa. We cultured cells in the charcoal-stripped FBS medium, which mimics ADT treatment in vitro and found that the intracellular glutamine levels were markedly increased with extension of ADT treatment in both cell lines (Fig. 1A, B), which is in line with our previous publication [15]. To investigate whether metabolite accumulation is a general phenomenon during ADT treatment, we performed a metabolite profiling but observed a global decrease of activity in multiple metabolic pathways, including glycolysis, the tricarboxylic acid (TCA) cycle, amino acid metabolism, nucleotide metabolism, and glutaminolysis (Fig. 1C), highlighting the unique distinction of glutamine from others. In agreement with the fact of attenuated glutaminolysis, the downstream metabolite of glutamine, glutamate, decreased slightly or largely remained unchanged after ADT treatment (Fig. S1A and B). We further calculated the ratio of glutamine/glutamate, which manifests the efficiency of glutamine catabolism, and found that the ratio was significantly increased (Fig. S1C and D). These results are consistent with our previous findings that glutamine utilization is blocked during ADT treatment [15]. Indeed, when we investigated the expression of GLS1, the key enzyme mediated the conversion from glutamine to glutamate, the results showed that ADT suppressed the expression of GLS1 (Fig. 1D, E, G, H). The intracellular glutamine pool dynamically consists of three major sources. In addition to the output of glutamine being used, the income of glutamine can be either absorbed from the outside cellular space or endogenously synthesized (Fig. 1F). To seek out whether the accumulated glutamine was contributed by glutamine uptake or glutamine synthesis pathways, we first examined the expression of the key enzymes. The expression of alanine-serine-cysteine transporter 2 (ASCT2), which is responsible for glutamine uptake, was dramatically decreased after ADT treatment (Fig. 1D, E, G, H). Consistently, when we measured glutamine concentration remained in the cell culture medium, which reflects the capability of cells for glutamine uptake, we found that the absorbed glutamine amount did not change too much, particularly at Day 7 and Day 14 (Fig. S1E and F), suggesting that the ability of glutamine obtaining for PCa cells is gradually attenuated during ADT treatment. Interestingly, the expression of GS, which is encoded by glutamate-ammonia ligase (GLUL) gene, was dramatically elevated at both mRNA and protein levels (Fig. 1D, E, G, H), indicating that the activity of glutamine synthesis might be enhanced triggered by ADT treatment. To corroborate this hypothesis, we further conducted ^13^C_6_-glucose isotope tracing experiments since the carbon backbone of endogenously synthesized glutamine is majorly derived from glucose. Although the glucose-labeled glutamine accounts for only ~10% of the total glutamine pool, ADT was able to elevate this proportion to ~15% (Fig. 1I, J). These results demonstrate that hormonal therapy upregulates the capability of glutamine anabolism in PCa cells.

To investigate whether ADT-induced glutamine accumulation can be also observed in vivo, we subcutaneously injected either LNCaP or LAPC4 cells in male mice to establish animal xenografts. When tumors reached ~100 mm^3^, we performed castration surgery by excising both testicles (Fig.. S1G). Xenograft tumors regressed markedly after castration (Fig.. S1H and I) and we harvested tumors for metabolome mass spectrometry analyses. In line with what we observed in vitro, castration led to a significant elevation of glutamine concentration (Fig. 1K, L) and the ratio of glutamine/glutamate (Fig. 1M, N), while left glutamate levels unchanged in tumors (Fig. S1J). Taken together, these data demonstrate that ADT suppresses the activity of glutamine catabolism and uptake pathways while enhances glutamine synthesis, which results in the accumulation of glutamine in PCa during hormonal therapy.

### Hormonal therapy-induced glutamine accumulation is due to the upregulation of GS expression

**Fig. 2:**
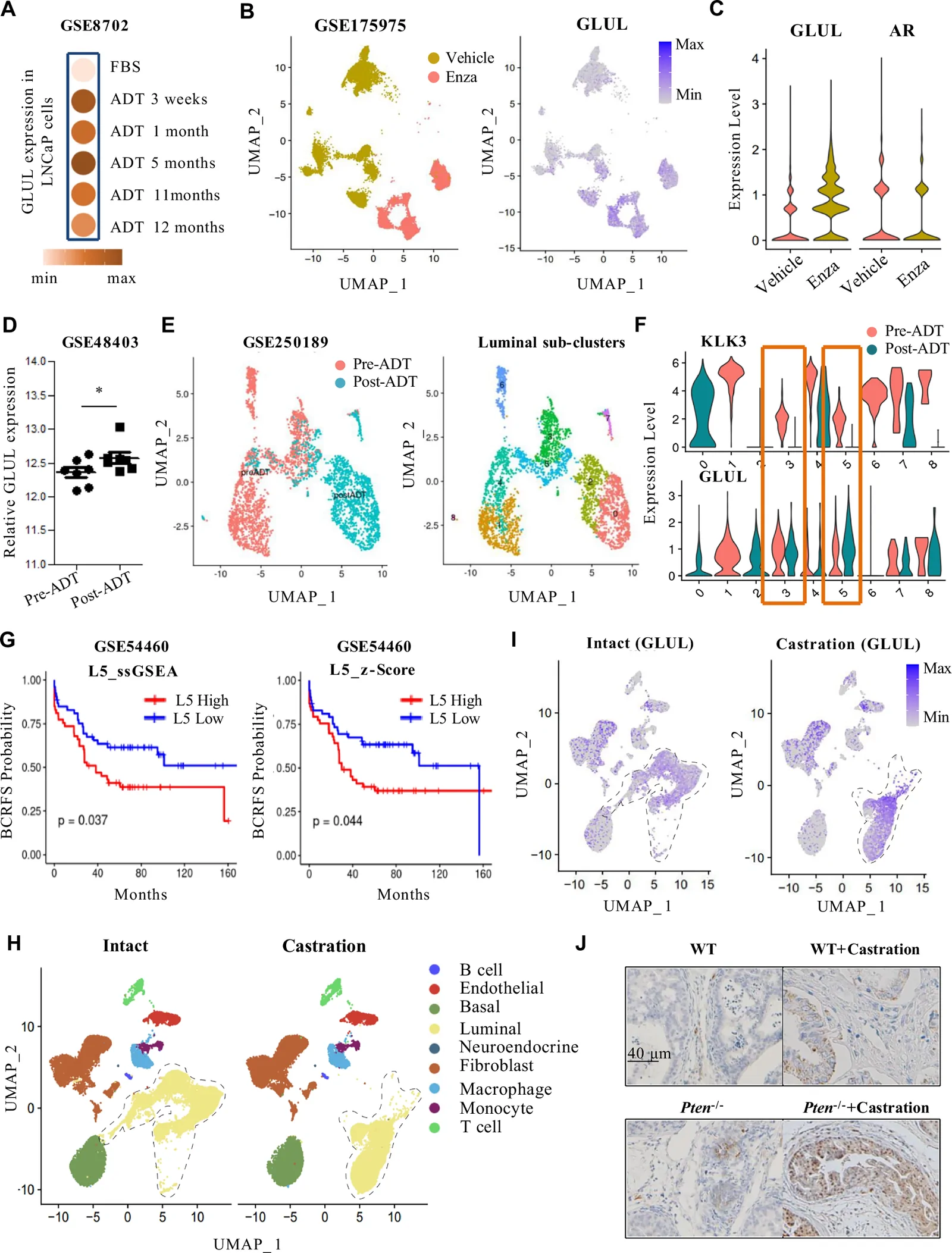
Hormonal therapy-induced glutamine accumulation is due to the upregulation of GS expression. **A** Dot plots showing the expression of GLUL after ADT at multiple time points. Data were retrieved from GSE8702. **B** Uniform manifold approximation and projection (UMAP) plots showing separation of LNCaP/AR cells treated by enzalutamide (Enza) (left) and GLUL enriched cell clusters (right). Data were retrieved from GSE175975. **C** Violin plots showing the overall expression of GLUL and AR in LNCaP/AR cells after Enza-treatment. **D** GLUL expression in patients pre- and post-ADT treatment. Data were retrieved from GSE48403. **E** Single-cell RNA sequencing data showing origin of luminal cells (left) and sub-clusters of luminal cells (right). Data were retrieved from GSE250189. **F** Violin plots showing KLK3 and GLUL expressions in luminal sub-clusters. **G** Kaplan-Meier survival plots showing PCa patients with low and high scores of L5 gene signature. **H** UMAP plots showing various cell clusters in mice treated with or without castration. Data were retrieved from GSE227109. **I** The projection of GLUL expression on the UMAP plots. **J** Immunohistochemical (IHC) staining of GS on prostate tissues from wild-type and *Pten*^*–/–*^ mice treated with or without castration. Scale bars were shown as indicated.

To verify whether the enhanced glutamine level during ADT treatment is due to the elevated expression of the key enzyme GS, we performed a comprehensive analysis across cell lines, patient samples, and animal models. First, the RNA-sequencing (RNA-seq) data of ADT treatment on LNCaP cells obtained from multiple time points (GSE8702) [18] showed that in comparison to the baseline level, the GLUL gene expression increased markedly along with androgen withdrawal (Fig. 2A). Similar results were also seen in a single-cell RNA-seq (scRNA-seq) dataset (GSE175975) [19]. Treatment of enzalutamide (Enza), a second generation of anti-androgen drug, on LNCaP/AR, which is an Enza-sensitive cell line and an ideal model for lineage plasticity studies, significantly separated cells into two distinct populations and GLUL expression was mainly enriched in the Enza-treated clusters (Fig. 2B), and the overall level was higher than that of the vehicle group (Fig. 2C). The expression of AR was set as a positive control. Second, we interrogated several patient sample-involved omics datasets. The GSE48403 database incorporated 7 paired patient samples obtained before and after ADT treatment [20]. Among those, only one patient exhibited decreased GLUL expression after hormonal therapy (Fig. S2A). The average level of GLUL showed significant elevation in post-ADT patient samples (Fig. 2D). We also analyzed four surgically obtained samples processed by scRNA-seq: two from patients who underwent one-month ADT treatment and two from treatment-naïve patients (GSE250189) [21]. Overall, the cellular composition remained comparable between pre-ADT and post-ADT groups, and the GLUL expression barely changed within luminal tumor cells (Fig. S2B and C). However, when we dissected luminal cells into more detailed sub-groups, we observed that cluster 3 (L3) and cluster 5 (L5) exhibited elevated GLUL expression yet decreased KLK3 levels (Fig. 2E, F). Interestingly, the position of L3 and L5 were in the middle between pre-ADT and post-ADT luminal cells, manifested by the uniform manifold approximation and projection (UMAP) visualization (Fig. 2E), suggesting that GLUL may play a pivotal role in the early stage of ADT to synthesize sufficient glutamine for treatment resistance and tumor recurrence. To find out the clinical relevance of those sub-luminal cells harboring high GLUL expression, we next retrieved unique genes to derive an L5-specific gene signature. High expression of the L5 signature was correlated with poorer biochemical recurrence-free survival (BCRFS), which was validated in two clinical cohorts [22, 23] by two algorithms (Fig. 2G and Fig. S2D). Lastly, we wanted to know whether the above observations can also be seen in animal models. The public scRNA-seq dataset [24] showed that castrating mice led to significant alterations of the luminal cell population while the tumor-surrounding cells were barely changed (Fig. 2H). More importantly, we noticed a portion of newly-emerged luminal cells that were strongly associated with the enrichment of GLUL gene expression (Fig. 2I). Quantification analysis verified the increased level of GLUL within the luminal cell cluster after mice being castrated (Fig. S2E). To examine the protein level of GS in tissues, we castrated *Pten* conditional knockout (*Pten*^*–/–*^) genetically-modified mice after PCa being spontaneously developed [25]. Immunohistochemical (IHC) staining of prostate tissues were subsequently performed and the GS expression was remarkably upregulated in the *Pten*-loss combined with castration tissue (Fig. 2J and Fig. S2F). In comparison, subtle expression changes were observed between wild-type *Pten* with and without castration groups (Fig. 2J and Fig. S2F). Together, these results showed that androgen withdrawal aberrantly boosts glutamine synthesis activity by upregulating the expression of the key enzyme, GS, which leads to a remarkable production of glutamine in PCa.

### Hormonal therapy-induced GS upregulation prevents the death of dormant cells

**Fig. 3:**
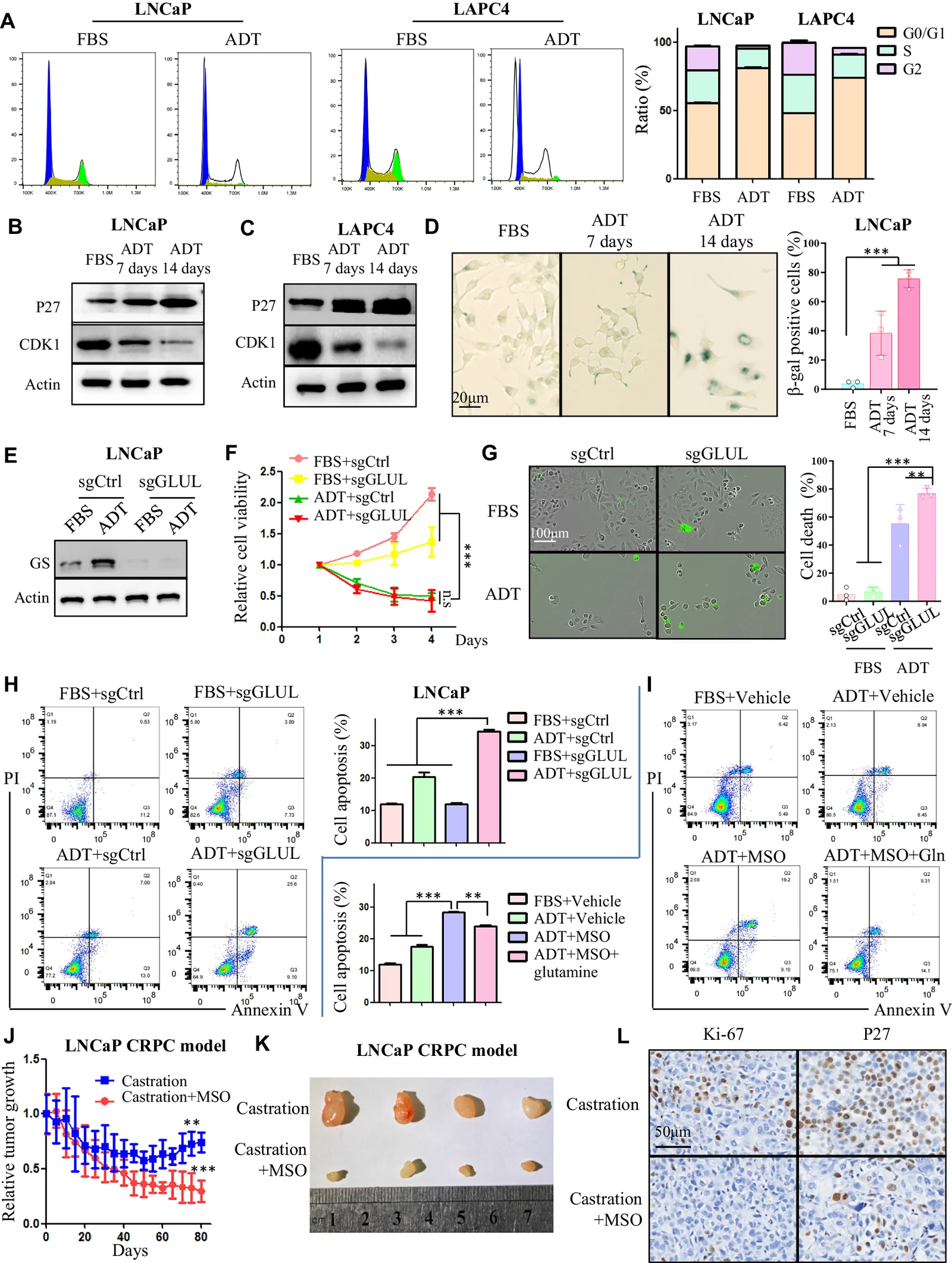
Hormonal therapy-induced GS upregulation prevents the death of dormant cells. **A** Flow cytometry showing cell cycle distribution of LNCaP and LAPC4 cells treated with ADT. **B**, **C** Immunoblots showing expression of P27 and CDK1 in LNCaP and LAPC4 cells treated with ADT. **D** β-staining showing representative images (left) and quantification (right) of positive cells. **E** Immunoblots showing loss of GS protein, achieved by CRISPR technique. **F** MTS assay showing cell viability in indicated treatment conditions. **G** Cytotox Green Dye staining showing dead cells which were positive for green dye. **H**, **I** Annexin V assay showing proportion of cell apoptosis in indicated conditions, determined by flow cytometry. **J**, **K** Relative tumor growth curve and tumor weight in LNCP xenografts, treated with castration alone or castration with MSO administration. **L** IHC staining showing expression of Ki-67 and P27. Data are depicted as mean ± s.d. *P < 0.05, **P < 0.01 and ***P < 0.001.

One of the possible reasons that tumor cells will inevitably recur after hormonal therapy is that androgen withdrawal is only able to lead tumor cells into dormant or quiescent status, rather than kill cells, and quiescence is reversible when ADT is removed [26]. We performed cell cycle analysis and noticed that ADT had most tumor cells arrested in G0/G1 phases (Fig. 3A). Additionally, we tested the expressions of two critical markers in PCa cells after ADT. Cyclin-dependent kinase 1 (CDK1) is a cell division regulatory protein that helps cells enter into S phase and mitosis and P27 is a classical marker for cell dormancy [27]. Immunoblots showed a considerable reduction of CDK1 after ADT treatment in both LNCaP and LAPC4 cells while the dormancy marker, P27, experienced a stepwise elevation along with androgen ablation (Fig. 3B, C). Additionally, β-gal staining, a measurement for cellular senescence, showed that ADT delayed cell growth determined by the increased proportion of senescent tumor cells (Fig. 3D and Fig. S3A).

Given the dramatic increase of intracellular glutamine levels, which is contradictory to the changes of other metabolites (Fig. 1A, C), we hypothesized that the upregulated GS might play a protective role for tumor cells mediating the ADT-induced cellular quiescence, instead of cell death. We employed the CRISPR-Cas9 technique to knock out GLUL in both LNCaP and LAPC4 cells. Immunoblots showed the efficiency of GS protein loss and it could no longer be induced by ADT treatment (Fig. 3E and Fig. S3B). Loss of GS slightly reduced cell proliferation rates in normal conditions. However, it barely affected cell proliferation ability in androgen-ablated environment (Fig. 3F and Fig. S3C), suggesting that GS suppression is not able to boost the efficacy of ADT treatment in slowing tumor cells’ growth. To determine whether GS can prevent tumor cells from death, we used Cytotox Green Dye to stain the nucleus of dead cells. GS-deficient groups exhibited higher percentages of green-positive dead cells in response to androgen withdrawal (Fig. 3G and Fig. S3D). Similarly, flow cytometry revealed that ADT combined with GS loss led to the highest proportion of apoptotic cells (Fig. 3H and Fig. S3E).

To verify the protective effect of GS in a pharmacological way, methionine sulfoximine (MSO), a potent inhibitor targeting GS, was employed. Enzymatic inhibition of GS abrogated the ADT-induced glutamine elevation (Fig. S3F). In line with genetic inhibition, the combined treatment of ADT and MSO led to the most significant reduction of cell survival (Fig. S3G). Supplementation of glutamine partially restored the survival of LNCaP and LAPC4 cells in response to the combined treatment of MSO and androgen withdrawal (Fig. S3G). Flow cytometry analysis further showed that the combined treatment of ADT and MSO achieved the most dramatic cell apoptosis, which could be modestly reversed by extra glutamine addition (Fig. 3I and Fig. S3H). These results demonstrate that GS prevents cell death of ADT-induced dormant cells. Suppression on GS might eradicate the ADT-induced cellular dormancy.

Next, we wondered whether disrupting dormant tumor cells by repression of glutamine synthesis could delay PCa relapse after ADT treatment. We have previously established a CRPC development animal model with LNCaP xenografts wherein tumor recurrence usually happens around 2 months after castration [15]. We then castrated male mice carrying LNCaP xenografts and recorded tumor volumes twice a week. We found that tumors regressed initially but kept growing after castration-alone for 8 weeks whereas the administration of MSO together with castration significantly delayed the onset of tumors developing into recurrence stages (Fig. 3J, K). IHC staining showed the decreased intensity of Ki-67 as well as P27 in the combined treatment groups (Fig. 3L and Fig. S3I), suggesting that suppressing GS results in more dormant cells to death and thereby prevents tumor recurrence with castration treatment.

### Transient upregulation of GS helps tumor cells overcome hormonal therapy

**Fig. 4:**
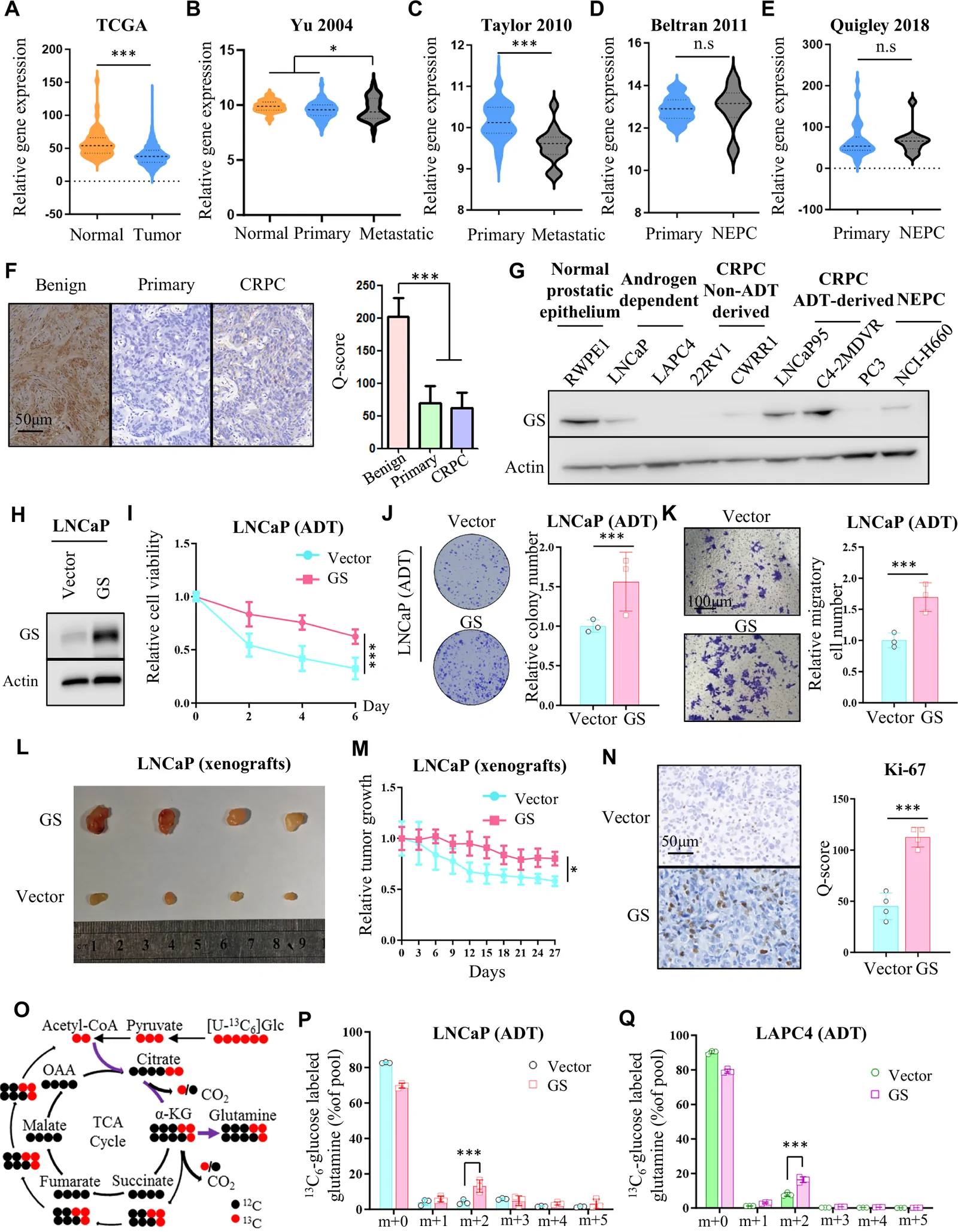
Transient upregulation of GS helps tumor cells overcome hormonal therapy. **A**–**E** Bioinformatics analysis derived from multiple datasets showing GLUL expression in normal prostate tissue, primary PCa tissue, and metastatic PCa/NEPC tissue. **F** IHC staining showing representative images (left) and quantification (right) of GS expression in benign prostate tissue, primary PCa tissue, and CRPC tissue. **G** Immunoblots showing GS expression in indicated cell lines. **H** Immunoblots showing GS expression in GS overexpressed cells. **I**–**K** MTS assay, colony formation assay and cell migration assay showing cell viability, clonogenicity, and migratory capability of GS overexpressed cells and vector cells in ADT conditions. **L**–**N** Representative images, relative tumor growth rate, and tumor weights of GS overexpressed xenografts and vector xenografts after castration. **O** IHC staining showing Ki-67 expression in GS overexpressed xenografts and vector xenografts after castration. **P** Schematic overview how glucose is involved in glutamine synthesis. OAA oxaloacetic acid. α-KG α-ketoglutarate. Glc glucose. **Q**–**R** Fractions of ^13^C_6_-glucose derived isotopologues in glutamine. Data are depicted as mean ± s.d. *P < 0.05, **P < 0.01 and ***P < 0.001.

Since almost every PCa patient invariably experiences the disease spectrum of initially ADT, then therapeutic failure and finally CRPC, we next asked whether the hormonal therapy-induced GS upregulation would be persistently at high levels in the late-stage of PCa. First, we compared GLUL expression between normal tissue and primary PCa tissue (Fig. 4A). It was not surprising that GLUL showed lower mRNA levels in primary PCa because glutamine synthesis pathway is not active in the hormone-sensitive stage, based on our previous studies [15, 16]. However, when we analyzed the GLUL expression in advanced PCa, such as metastatic PCa, CRPC, or neuroendocrine PCa (NEPC, a histologically lethal variant of PCa), we found that the GLUL expression was not increased or even lower than that in primary PCa tissue (Fig. 4B). This observation was validated in multiple public sequencing datasets (Fig. 4C–E). IHC staining of GS showed a similar expression pattern. Primary PCa and CRPC patient tissues had comparable GS expressions, both of which were weaker than that in normal prostate tissues (Fig. 4F). Additionally, we employed a panel of PCa cell lines, which represent different clinical background, to examine the GLUL expressiion and surprisingly observed that only cells derived from anti-androgen treatment (e.g C4-2MDVR and LNCaP95, derived from long-term treatment of enzalutamide on C4-2 and LNCaP cells, respectively) had evident expression of GS (Fig. 4G), whereas others, regardless of primary PCa cells (e.g LNCaP and LAPC4) or cells representing the terminal-stage of PCa (e.g PC3 and NCI-H660), had little expression of GS (Fig. 4G). These results raise a highly possibility that GS is upregulated in a transient period particularly at the initial stage of hormonal therapy and then decreases when tumor cells develop into advanced stages.

Although the duration of GS upregulation is relatively short, it still might mediate resistance of PCa cells to hormonal therapy. Indeed, overexpression of GS in LNCaP and LAPC4 cells considerably increased cell viability and enhanced tumor cell’s ability of colony formation and migration, even under the androgen-deprived condition (Fig. 4H–K and Fig. S4A–D). Xenograft models generated by LNCaP and LAPC4 cells also showed that GS-overexpressed tumors were more resistant to castration (Fig. 4L–M and Fig. S4E-F). IHC staining of Ki-67 for the dissected tumors showed a higher intensity in the GS-overexpressed groups (Fig. 4N and Fig. S4G), indicating that tumor cells are more proliferative with GS overexpression.

To further determine that the therapeutic resistance is through the glutamine synthesis pathway, we performed metabolome analyses. Intracellular glutamine levels were increased after GS was ectopically overexpressed, accompanied with decreased levels of glutamate (Fig. S4H–K), indicating that the activity of glutamine synthesis is upregulated. The ^13^C_6_-glucose isotopomer tracing analysis was then performed to determine the efficiency of glutamine synthesis. The contribution of glucose to the carbon backbone of glutamine is generally via the TCA cycle. Glucose (m + 6) breaks down into pyruvate (m + 3), acetyl-CoA (m + 2) and then enters the TCA cycle, condensating with oxaloacetic acid (OAA, m + 0) to form citrate (m + 2). Citrate gets rid of one carbon to generate m + 1 or m + 2 α-KG and glutamine, depending on whether the disposed carbon is from labeled glucose or not. As the cycle runs, m + 3, m + 4 and m + 5 glutamine are subsequently produced by continuously assimilating isotopomer-labeled acetyl-CoA (Fig. 4O). Overall, in ADT conditions, GS overexpression led to the increased proportion of glucose-labeled citrate, α-KG, and glutamine. By contrast, the percentage between labeled and unlabeled succinate, fumarate, and malate was not remarkably affected by such genetic changes (Fig. S4L and M). Furthermore, in comparison to other isotopomer-labeled glutamine, the m + 2 glutamine was the most significantly increased metabolite among the metabolite pool after GS was overexpressed (Fig. 4P, Q). Similar expression pattern was also seen in citrate and α-KG (Fig. S4N-Q), indicating that overexpressing GS partially drives the TCA cycle’s activity (particularly at the first cycle where m + 2 metabolites are generated) to facilitate the production of glutamine (Fig. 4O, highlighted by the purple arrow). Taken together, these results suggest that ADT temporarily induces GS upregulation to help tumor cells resist to hormonal therapy by producing sufficient glutamine. However, when tumors overcome ADT treatment and become terminal-stage PCa (such as CRPC and NEPC), the GS levels drop to the baseline since the capability of glutamine uptake is restored or even enhanced in tumor cells [28].

### GLUL is transcriptionally repressed by AR

**Fig. 5:**
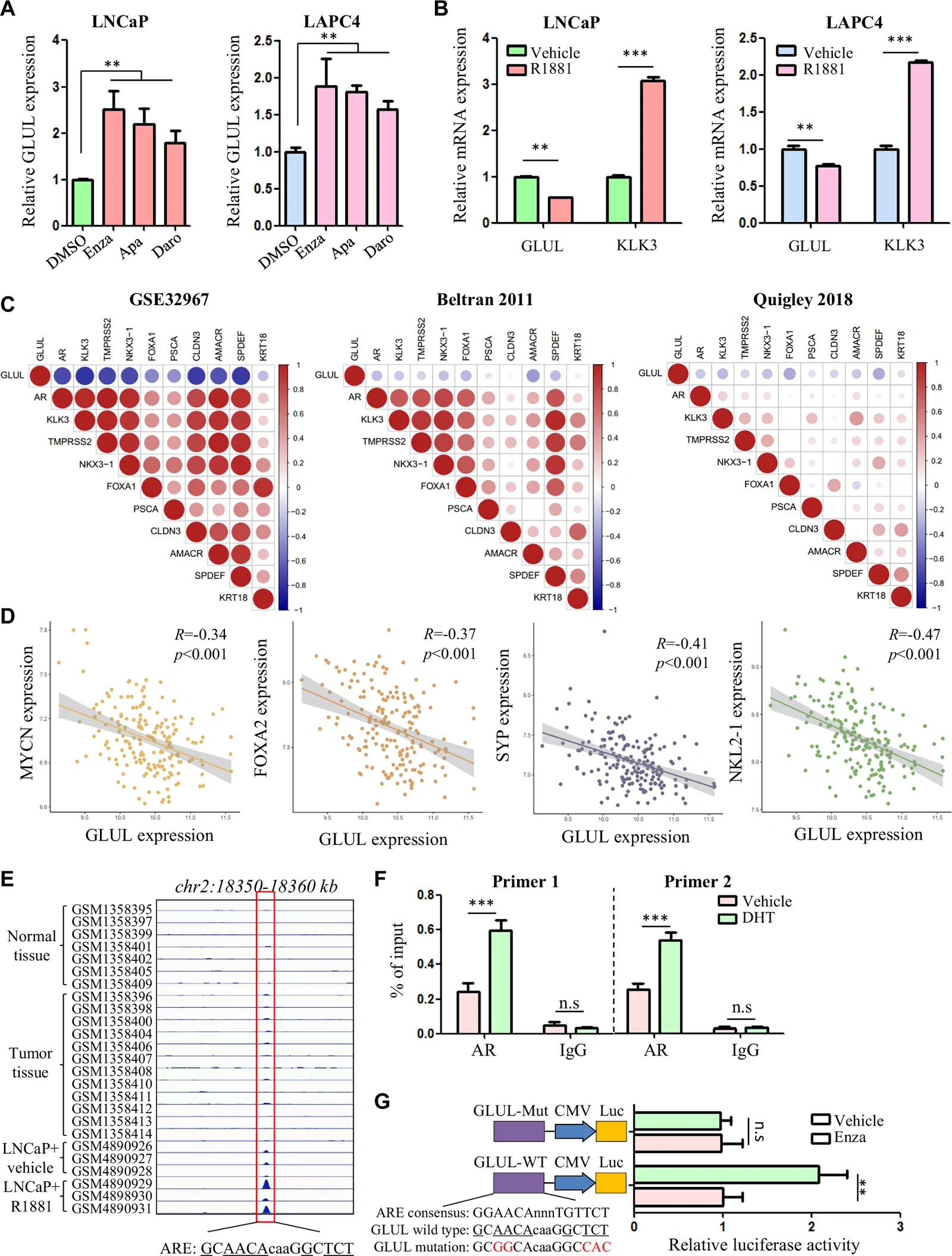
GLUL is transcriptionally repressed by AR. **A** Transcript levels of GLUL in LNCaP and LAPC4 cells treated with enzalutamide (Enza), apalutamide (Apa), and darolutamide (Daro), respectively. **B** Transcript levels of GLUL in LNCaP and LAPC4 cells treated with R1881. **C** Heatmap showing correlation of GLUL with AR-related genes from multiple public datasets. **D** Correlation analysis showing negative association of GLUL with NE-related markers. Data was retrieved from GSE21034. **E** Genomic browser representation of AR binding in GLUL intron 6 encompassing an ARE. Nucleotides identical to the canonical ARE consensus were underlined. **F** ChIP-qPCR showing AR binding to GLUL intron 6 upon dihydrotestosterone (DHT) stimulation, verified by two sets of primers. **G** Luciferase activity of modified constructs containing wild type (WT) GLUL intron 6 or mutant (Mut) GLUL intron 6 treated by enzalutamide (Enza). Schematic of luciferase reporter constructs were shown on the left. Data are depicted as mean ± s.d. * *P* < 0.05, ** *P* < 0.01 and ***P < 0.001.

The significant impact of AR inhibition on GS expression implies the possibility that GLUL is an AR-regulated gene. Indeed, the mRNA level of GLUL was pronouncedly elevated by AR repression, which was validated by multiple AR inhibitors (enzalutamide, darolutamide, and apalutamide) (Fig. 5A). Conversely, the AR stimulator, R1881, markedly led to decrease of GLUL expression at both mRNA and protein levels (Fig. 5B and Fig. S5A), suggesting that GLUL is negatively regulated by AR. To further corroborate the inverse correlation between GLUL and the AR signaling pathway, we interrogated a panel of clinical cohorts [29–31]. Bioinformatics analysis showed that GLUL was negatively associated with AR-related genes, including KLK3, TMPRSS2, NKX3-1, FOXA1 (Fig. 5C). Interestingly, GLUL was not positively correlated with NE markers (Fig. 5D), which again indicates that GLUL does not persistently express in advanced PCa, such as treatment-induced NEPC, after hormonal therapy.

To explore whether AR binds to the GLUL gene locus, we analyzed the chromatin immunoprecipitation sequencing (ChIP-seq) dataset [32, 33] and found that in the presence of R1881, AR showed a significant peak at the sixth intron of GLUL in LNCaP cells (Fig. 5E). In clinical samples, higher AR occupancy at intron 6 was shown in most tumor tissues in comparison to normal prostate tissues (Fig. 5E). Furthermore, we identified a putative androgen response element (ARE) within the AR-bound intronic sequence. ChIP-qPCR assays verified the occupancy of AR at this ARE site by two different paired primers (Fig. 5F). To determine whether the ARE was functional, we constructed the luciferase reporters by cloning the ARE sequences into luciferase plasmids (wide-type) and mutated selected-nucleotides (mutant) (Fig. 5G). The activity of luciferase increased significantly upon enzalutamide administration while remained unchanged in the mutant constructors (Fig. 5G). These results suggest that androgen-activated AR binds to the intronic DNA of GLUL and suppresses its transcription whereas androgen deprivation releases this suppression and leads to GLUL transcription.

## Discussion

**Fig. 6:**
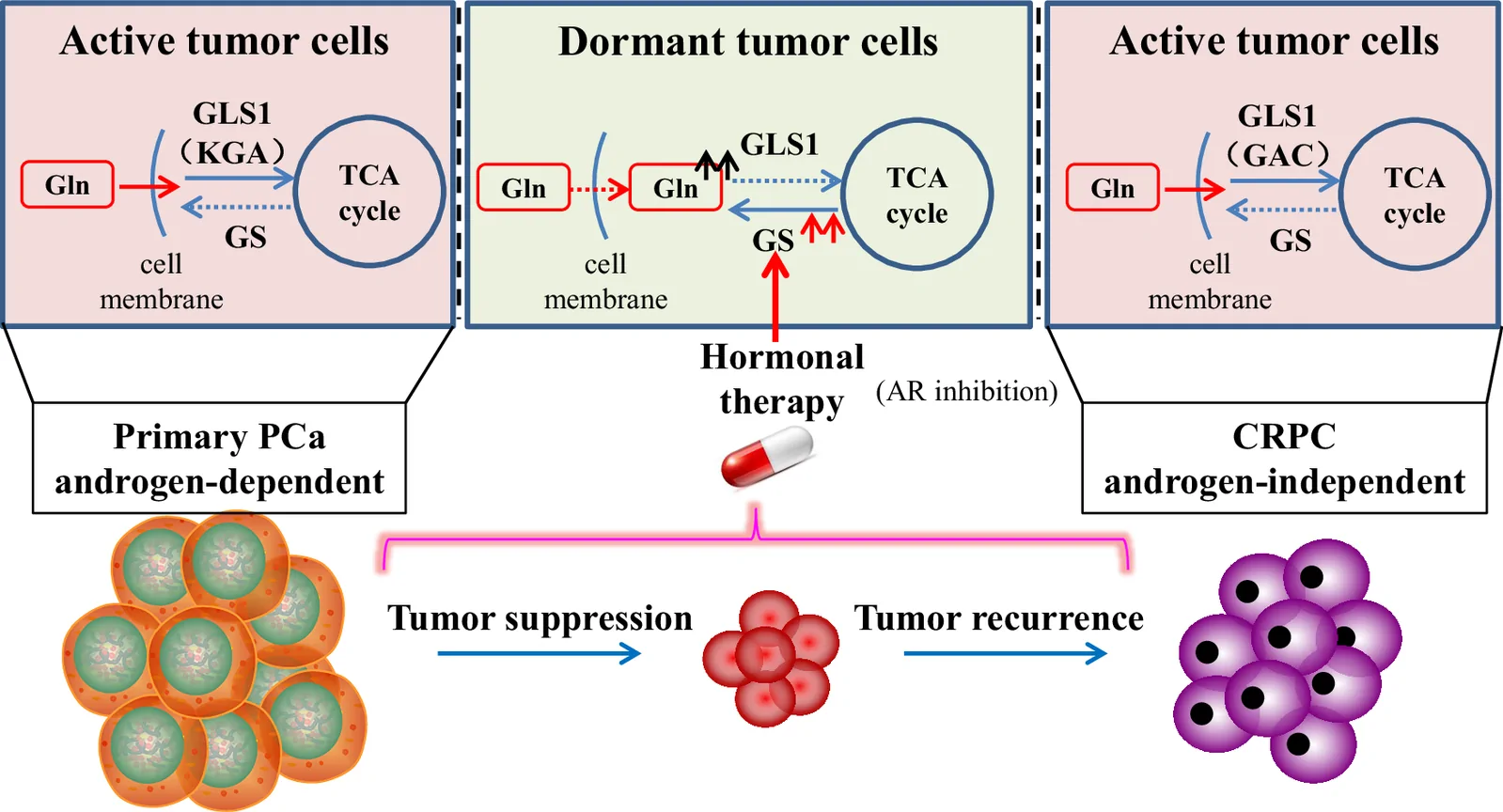
Diagram of proposed mechanism that how dormant prostate cancer cells escape hormonal therapy via upregulating the glutamine synthesis pathway.

In this study, together with our previous publications, we elucidate a plausible explanation why PCa patients invariably relapse after hormonal therapy based on a metabolic foundation. Generally, in primary androgen-dependent PCa, glutamine can be sufficiently transported into tumor cells and catalyzed by the kidney-type glutaminase (KGA) isoform of GLS1 to drive the TCA cycle. Hormonal therapy administration leads PCa cells into dormancy and meanwhile shuts down glutamine uptake and glutamine utilization pathways. Of note, since GS escapes AR suppression due to AR-inhibited treatment, the endogenous glutamine synthesis pathway is highly active. Upregulated glutamine anabolism and downregulated glutamine catabolism coordinately result in glutamine accumulation in tumor cells. When glutaminase C (GAC), another isoform of GLS1, predominates in tumor, the previously stored glutamine becomes readily energy source to help tumor cells overcome hormonal therapy and switch the nutritious dependence of tumor cells from androgen to glutamine. After disease progresses into castration-resistant stages, tumor cells are able to efficiently obtain extracellular glutamine since glutamine uptake pathway is recovered. As a result, the function of glutamine synthesis pathway is greatly attenuated. Glutaminolysis becomes the most vital pathway to support cell proliferation and aggressiveness of CRPC (Fig. 6).

The glutamine synthesis pathway usually functions as a crucial mechanism for eliminating free amines, which are harmful substances and pose a threat to cellular homeostasis. The two-steps biosynthetic process from α-KG to glutamine assimilates two molecules of free amines, thereby effectively scavenging excess free amines and mitigating their cytotoxicity. Of particular interest, accumulating evidence has revealed the role of glutamine synthesis in tumor contexts. For instance, in hepatocellular carcinoma (HCC), this pathway mediates Yes-associated protein (YAP)-induced HCC development through downstream regulatory cascades [34]. Consistently, studies using murine HCC models have confirmed that β-catenin can enhance the activity of glutamine synthesis, which in turn upregulates the mTORC1 signaling pathway, a critical driver of cell proliferation and survival, ultimately facilitating HCC progression [35].

The activity of glutamine synthesis is largely relied on the rate-limiting enzyme GS. In addition to HCC, GS also plays pivotal roles in driving tumor development in multiple solid tumors, including glioma, luminal breast cancer, non-small cell lung cancer (NSCLC), and pancreatic ductal adenocarcinoma (PDAC) [36–38]. Notably, these GS-highly expressed tumors share several common metabolic characteristics that underscore their reliance on de novo glutamine synthesis. Firstly, most of those cancer types are poorly vascularized, which impairs the uptake of extracellular glutamine from the systemic circulation. This metabolic constraint is particularly prominent in PDAC, where tumor cell-surrounded stroma further limits nutrient availability [38]. Secondly, these tumors exhibit GLS1 independence, meaning that inhibition on glutaminolysis exerts minimal suppressive effects on their survival and proliferation. Thirdly, depletion of extracellular glutamine does not reduce the activity of the TCA cycle or the levels of its associated metabolites, indicating that mitochondrial function and TCA cycle homeostasis are no longer dependent on exogenous glutamine. These features emphasize the pathological and biological significance of GS and the glutamine synthesis pathway in tumorigenesis.

Intriguingly, the function of glutamine synthesis and GS in PCa is different. In comparison to those tumors mentioned above, the endogenous level of GS is relatively low in both primary and advanced PCa, revealed by public datasets, human tissues and in vitro models. However, this fact does not mask the importance of GS in disease progression, particularly in hormonal therapy resistance. We believe that GS predominantly functions as a “rescue factor” that aids tumor cells in overcoming anti-androgen managements. We observe that hormonal therapy induces GS upregulation and increased glutamine production in patient sequencing data and validated in both cell and animal models. Additionally, glucose-tracing experiments reveal that most carbon atoms in glutamine are labeled by glucose, and the proportion of labeled glutamine was markedly increased after ADT treatment, which indicates an elevation of glutamine synthesis activity. More importantly, ectopically overexpressing GS enhances tumor viability and decreases cell apoptosis in androgen-deprived environment. These results suggest that GS is actually not an essential component in either early hormone-dependent PCa or advanced CRPC, but indispensable for mediating resistance to hormonal therapy. The biological phenomenon that GS transiently elevates during hormonal therapy but decreases after recurrence can be attributed to several potential reasons. First, we identify that GS is negatively regulated by AR. To this end, since 80 ~ 90% CRPC patients harbor AR overexpression/amplification, activating AR mutations and constitutively active AR splice variants, it is not surprising to observe the re-suppression of GS by AR after tumor overcomes hormonal therapy [39]. Second, our previous publications demonstrate that advanced PCa, including CRPC and NEPC, heavily relies on glutamine uptake and glutamine catabolism pathways with high expression of the enzymes, ASCT2 and GLS1 [15, 16]. These facts reveal that advanced PCa is able to obtain glutamine from extracellular space instead of the intracellular glutamine synthesis pathway that may no longer need the overexpression of GS.

Tumor resistance to hormonal therapy remains a long-lasting issue in PCa management. Cellular dormancy is widely recognized as a key contributor to biochemical recurrence following hormonal therapy for PCa. This dormant state can, on one hand, suppress the growth of primary tumors, creating an illusion of “complete clinical remission” for clinicians, but on the other hand, enable tumor cells to undergo distant metastasis silently without being detected. Previous studies report that exosome-derived NR2F1 serves as a critical factor driving the transition of PCa cells from dormancy to proliferation [40]. Similarly, transcription factor PRDM16 has been demonstrated to initiate the dormancy program and promote bone metastasis of tumor cells [41]. In addition, the switch from AR to glucocorticoid receptor (GR) can protect dormant tumor cells from apoptosis via the tryptophan 2,3-dioxygenase (TDO2) -kynurenine (Kyn) -aryl hydrocarbon receptor (AhR) pathway during hormonal therapy [42]. In this study, we provide a novel insight into the mechanism of PCa cell dormancy from a metabolic perspective. Hormonal therapy inhibits AR activity, leading to cell dormancy, while concurrently upregulating the GS expression. The elevated GS synthesizes substantial amounts of glutamine, which serves as a reserve for subsequent tumor recurrence. Inhibiting GS activity in combination with hormonal therapy can effectively cut off the metabolic substrates required for tumor recurrence, thereby significantly prolonging the response duration of tumors to hormonal therapy. Collectively, our study may suggest that the combination of hormonal therapy and GS inhibitors may significantly reduce the biochemical recurrence possibility of PCa, offering substantial clinical benefits to patients with advanced PCa.

## Materials and methods

### Cell culture

LNCaP, PC3, C4-2, C4-2MDVR, 22RV1, CWRR1, and DU145 cells were purchased from ATCC. All cell lines were identified by STR profiling. LNCaP, PC3, C4-2, C4-2MDVR, 22RV1, CWRR1, and DU145 cells were maintained in RPMI 1640 medium (Gibco), NCI-H660 cells were cultured in RPMI 1640 medium with 1% insulin transferrin selenium, 0.02‰ hydrocortisone and 0.02‰ β-estradiol. LAPC4 cells were maintained in IMDM medium (Gibco). RWPE1 cells, which represent normal prostate epithelium, were cultured in keratinocyte serum-free medium (Gibco). All media were supplemented with 10% fetal bovine serum (FBS) (Corning) and 1% penicillin-streptomycin (Gibco). Androgen-deprived therapy (ADT) in LNCaP and LAPC4 cells was carried out by using RPMI 1640 (Gibco, phenol red free) and IMDM (Gibco, phenol red free), respectively, supplemented with 10% charcoal dextran stripped fetal bovine serum (CS-FBS) (Denville) and 1% penicillin-streptomycin. LNCaP95 cells were cultured in RPMI 1640 (Gibco) with 10% CS-FBS. C4-2MDVR and LNCaP95 cells were maintained with 10 μM enzalutamide.

### Cell viability assay

1000 cells/well were plated in 96-well plates. For the androgen deprivation experiments, either complete medium or CS-FBS medium was used for cell culture. MTS assay (Biovision) kit was performed to measure cell viability at the indicated time points per manufacturer’s protocol. For the Cytotox Green staining experiments, the specific green dye was added to the cell culture medium and green fluorescence will be monitored by the Incucyte equipment when cells become dead or unhealthy.

### Glutamine and glutamate concentration measurement

The procedure was performed based on manufacture’s instruction (Promega). Basically, for each prepared sample, 25 μl aliquots mixed with glutamine/glutamate controls was transferred into a white, 96-well luminometer plate. 25 μl glutaminase enzyme solution was added. After shaking the plate for 60 s and 30 min incubation, 50 μl glutamine/glutamate detection reagent was added. A plate-reading luminometer was used to record luminescence.

### Metabolite measurement of tumors by mass spectrometry

The tumor tissues were weighed and placed into centrifuge tubes, then immersed in liquid nitrogen for 2 min. After removal, the tissues were wrapped in aluminum foil and returned to liquid nitrogen for approximately 1 min. The tissues were then pulverized as thoroughly as possible. High performance liquid chromatography (HPLC)-grade 80% methanol was added to ensure thorough mixing of the tissue with the solution. The mixture was left to stand on ice for 10 min, followed by centrifugation at 4 °C for 10 min. The supernatant was transferred to a new 1.5 ml eppendorf tube and subjected to a vacuum centrifugation until crystalline material was visible at the bottom of the tube. The samples were subsequently submitted for mass spectrometry analysis to determine metabolite concentrations.

### ^13^C_6_-glucose tracing experiments

Cells were seeded in 6-well plates. Medium containing ^13^C_6_-glucose isotope was added to replace the original culture conditions. After 24 h incubation, medium was aspirated and 80% pre-chilled HPLC-grade methanol was added to each well. Adherent cells were then scraped into the solvent and the supernatant was extracted after cell pellets and debris were spun down. Solvent was further dried with a speed vacuum and dry pellets were collected. Samples were submitted for mass spectrometry analysis.

### Immunohistochemical staining

Xenograft sections and human tissue blocks were deparaffinized, rehydrated, and boiled in citrate buffer (pH = 6.0) for 40 min. Antibodies were incubated for 1 h followed by incubation of secondary antibody. Diaminobenzidine (DAB) was then applied for visualization. Quick-score system was performed in all the immunohistochemical staining tissues. The intensity of staining is multiplied by the percentage of staining to derive a composite score (a range from 0 to 300). Primary antibodies used are as followings: anti-GS (1:100, Thermo Fisher, PA5-28940), anti-P27 (1:300, Cell Signaling Technology, #2552), anti-Ki-67 (1:300, Thermo Fisher, 740008 T).

### Gene silencing and overexpression

The lentiCRISPRv2 plasmid was purchased from Addgene (52961). The sgRNA of GLUL was designed according to the Target Guide Sequence Cloning Protocol of Zhang Feng’s laboratory. The Oligos are as follows:

GLUL-sgRNA-Forward: 5′ CACCGGAGCCATCGAAATTCCACTC 3′

GLUL-sgRNA-Reverse: 5′ AAACGAGTGGAATTTCGATGGCTCC 3′

Lentiviral plasmids were packaged into lentivirus and infected into cells, followed by puromycin (2 μg/ml) selection. Puromycin-resistant cells were pooled, amplified and analysis. Protein knockdowns were confirmed by western blotting. For GLUL overexpression, ORF plasmids were purchased from Origene (RC204039). Overexpressed GS was confirmed by western blot.

### RNA extraction and quantitative real-time PCR

Total RNA was extracted using RNA extraction kits (Fastagen, China). The PrimeScript RT Master Mix (Takara) was used for cDNA synthesis. Quantitative real-time PCR (qRT-PCR) was performed on ABI 7500 (Applied Biosystems) using SYBR Green PCR Master Mix (Quanta Biosciences). Actin expression assay was used as an internal control.

Primer sequences:

GLS1-Forward: 5′ TGCATTCCTGTGGCATGTAT 3′

GLS1-Reverse: 5′ TTGCCCATCTTATCCAGAGG 3′

ASCT2-Forward: 5′ CATCATCCTCGAAGCAGTCA 3′

ASCT2-Reverse: 5′ CTCCGTACGGTCCACGTAAT 3′

KLK3-Forward: 5′ GATGAAACAGGCTGTGCCG 3′

KLK3-Reverse: 5′ CCTCACAGCTACCCACTGCA 3′

GLUL-Forward: 5′ CTGCCATACCAACTTCAGCACC 3′

GLUL-Reverse: 5′ AATAGGCACGGATGTGGTACTGG 3′

Actin-Forward: 5′ AGAGCTACGAGCTGCCTGAC 3′

Actin-Reverse: 5′ AGCACTGTGTTGGCGTACAG 3′

### Immunoblotting

Total protein lysate was collected by lysing adherent cells with RIPA buffer supplemented with phosphatase and protease inhibitor cocktail. Lysates were cleared by centrifugation and protein concentration in supernatant was determined by the BCA assay kit. Equal amounts of total protein were used for immunoblotting. After electrophoresis, the proteins were transferred to polyvinylidene difuoride transfer membrane (PVDF) followed by 5% non-fat milk blocking and incubated with primary antibodies. After washing with TBST (TBS with 0.1% Tween), corresponding secondary antibodies conjugated with horseradish peroxidase-conjugated (HRP) were used to incubate the membranes. Samples were developed by Chemiluminescent Substrate (Thermo Fisher) and exposed by Odyssey Imaging Systems (LI-COR). Primary antibodies are used as followings: anti-GS (1:500, Thermo Fisher, PA5-28940), anti-GLS1 (1:500, Proteintech, 29519-1-AP), anti-ASCT2 (1:1000, Cell Signaling Technology, #5345), anti-P27 (1:1000, Cell Signaling Technology, #2552), anti-CDK1 (1:2000, Proteintech, 10762-1-AP), anti-actin (1:4000, Proteintech, 20536-1-AP).

### Animal experiments

Three to four-week-old male BALB/C nude mice were purchased from Jicui Yaokang Biotechnology (Nanjing, China). 10^7^ cells were injected subcutaneously in male NSG mice. When tumors reached around 150 mm^3^, mice were randomly either received orchiectomy or sham treatment (four mice for each condition group). Tumor growth was followed by caliper measurement twice a week or every three other days. Volumes of individual tumors were normalized to those on day 0 (day of castration). Tumors were harvested after mice were euthanized. For MSO treatment combined with castration, 10 mg/kg of MSO was administrated via intraperitoneal injection [37]. Harvested tumors were either embedded into paraffin followed by hematoxylin and eosin staining and immunohistochemistry staining or prepared for mass spectrometry analysis. Animal studies were performed in accordance with institutional guidelines approved by Anhui Medical University.

### ChIP-qPCR

The cells were crosslinked with 1% formaldehyde for 10 min at room temperature, followed by 125 mM glycine addition to stop the reaction. Cells were washed with cold PBS and re-suspended in cell lysis buffer (1% SDS, 1 mM EDTA, and 25 mM Tris–HCl, pH 8.0) for 30 min at 4 °C. Supernatant was obtained by centrifuge. Sonication was used to shear the chromatin to lengths between 100 and 500 bp. The protein‒DNA complexes were immunoprecipitated with either IgG or AR antibodies, followed by further incubation with protein magnetic beads for 2 h at 4 °C. The complexes were washed with low-salt, high-salt and LiCl wash buffers sequentially, followed by two washes with Tris-EDTA buffer at 4 °C. The Tris-EDTA complex was eluted by adding 100 μL of elution buffer (1% SDS, 0.1 M NaHCO3). NaCl (0.2 M) and proteinase K (0.5 mg/mL) were used to reverse crosslinking, and the mixture was incubated at 65 °C overnight. DNA was purified with a DNA purification kit. The purified DNA was dissolved in ddH2O for qPCR. The sequences of the primers used for ChIP‒qPCR are listed below.

GLUL-1-Forward: 5′ CAACACAAGGCTCTTGGTAGC 3′

GLUL-1-Reverse: 5′ CTTCGTGGGAGTGTACTGTGG 3′

GLUL-2-Forward: 5′ AGGCTCTTGGTAGCCTGATT 3′

GLUL-2-Reverse: 5′ ACTTCGTGGGAGTGTACTGT 3′

### Statistical analysis

All experiments in this study were performed with at least three independent replicates. All quantitative results are displayed as the mean ± SD. For normally distributed data, the statistical difference between two groups was compared using a Student’s *t* test. If more than two groups were compared, ANOVA was used. Homogeneity of variances between compared groups was tested prior to parametric comparisons. Statistical analysis was determined using Prism 5 software (GraphPad Software, Inc.). A *p* value of less than 0.05 was considered statistically significant.

## Supplementary information

Supplemental figures

Uncropped WB

## Data Availability

Data cohorts were retrieved from the Cancer Genome Atlas (TCGA), Gene Expression Omnibus (GEO) and cBioPortal. Metabolic pathway enrichment analysis was achieved by MetaboAnalyst software. All codes for data process are available upon reasonable requests.
