# Supplementary material for: Glutamine synthesis pathway promotes the survival and recurrence of dormant tumor cells in prostate cancer
Source: Cell Death Discov. 2026 Aug 27;12:420. doi: 10.1038/s41420-026-03282-w (PMC13683334; doi:10.1038/s41420-026-03282-w)
Supplement: Supplementary file 1 [file 41420_2026_3282_MOESM1_ESM.pdf]

Fig. S1

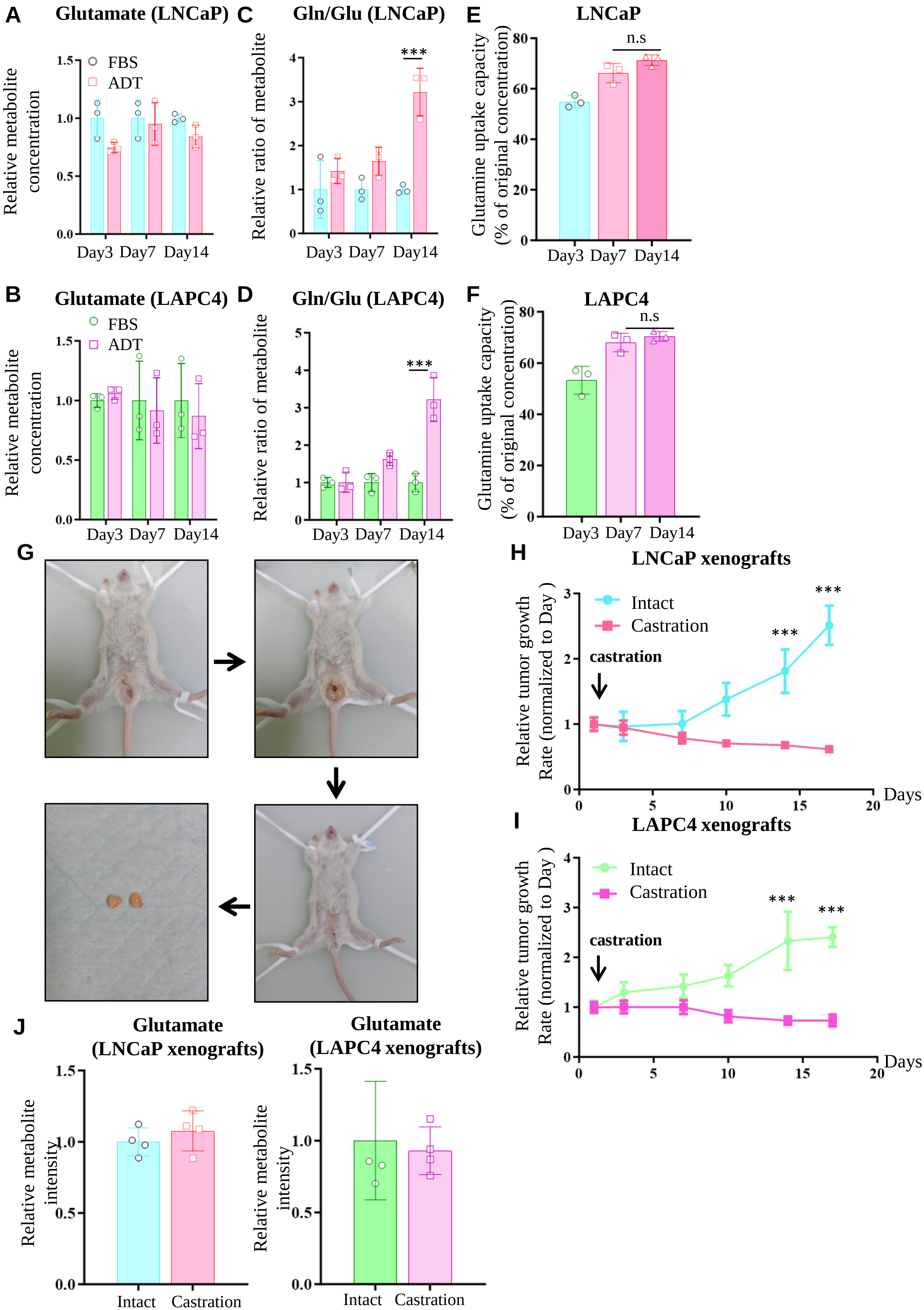

A
Glutamate (LNCaP)
FBS
ADT
Relative metabolite concentration
1.5
1.0
0.5
0.0
Day3 Day7 Day14
C
Gln/Glu (LNCaP)
Relative ratio of metabolite
***
4
3
2
1
0
Day3 Day7 Day14
E
LNCaP
Glutamine uptake capacity (% of original concentration)
n.s
80
60
40
20
0
Day3 Day7 Day14
B
Glutamate (LAPC4)
FBS
ADT
Relative metabolite concentration
1.5
1.0
0.5
0.0
Day3 Day7 Day14
D
Gln/Glu (LAPC4)
Relative ratio of metabolite
***
5
4
3
2
1
0
Day3 Day7 Day14
F
LAPC4
Glutamine uptake capacity (% of original concentration)
n.s
80
60
40
20
0
Day3 Day7 Day14
G
H
LNCaP xenografts
Relative tumor growth Rate (normalized to Day)
Intact
Castration
castration
***
***
3
2
1
0
0 5 10 15 20
Days
I
LAPC4 xenografts
Relative tumor growth Rate (normalized to Day)
Intact
Castration
castration
***
***
4
3
2
1
0
0 5 10 15 20
Days
J
Glutamate (LNCaP xenografts)
Relative metabolite intensity
1.5
1.0
0.5
0.0
Intact Castration
Glutamate (LAPC4 xenografts)
Relative metabolite intensity
1.5
1.0
0.5
0.0
Intact Castration

(A and B) Intracellular glutamate levels measured in LNCaP or LAPC4 cells treated with either FBS or ADT at indicated time points. (C and D) The ratio of glutamine (Gln)/glutamate (Glu) calculated in LNCaP and LAPC4 cells. (E and F) The change of glutamine uptake capacity of LNCaP and LAPC4 cells treated with ADT. Glutamine uptake capacity was determined by the percentage of remained glutamine in the original culture medium. (G) Representative images showing the surgical procedure of orchiectomy in mice. (H and I) Tumor growth curves of LNCaP and LAPC4 xenografts after surgical castration. (J) LC-MS showing relative glutamate intensity in LNCaP and LAPC4 xenografts after castration. Data are depicted as mean ± s.d. * $P < 0.05$, ** $P < 0.01$ and ***$P < 0.001$.

Fig. S2

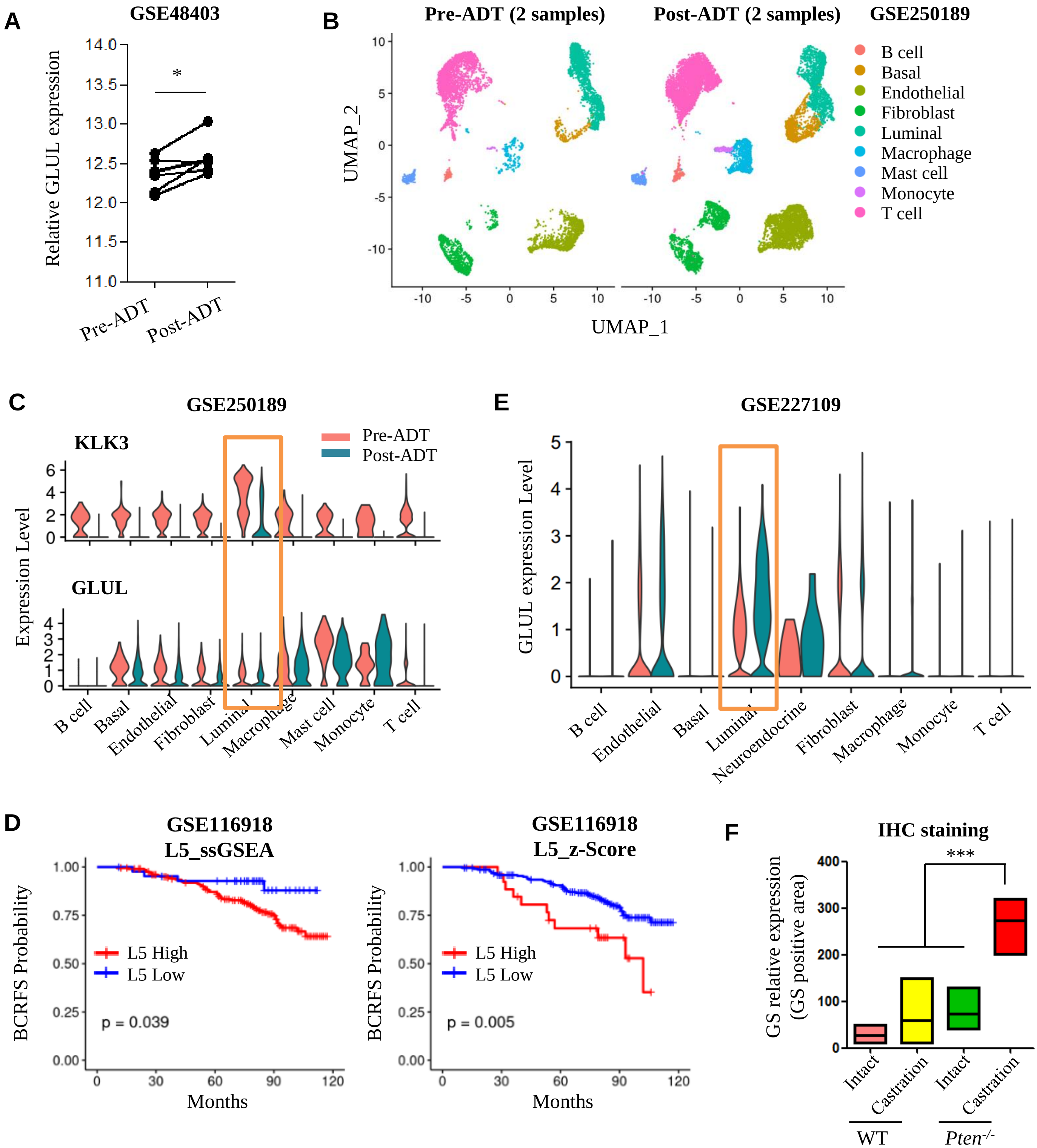
A
GSE48403
Relative GLUL expression
Pre-ADT
Post-ADT
*
B
Pre-ADT (2 samples)
Post-ADT (2 samples)
GSE250189
UMAP_2
UMAP_1
B cell
Basal
Endothelial
Fibroblast
Luminal
Macrophage
Mast cell
Monocyte
T cell
C
GSE250189
KLK3
GLUL
Pre-ADT
Post-ADT
Expression Level
E
GSE227109
GLUL expression Level
Neuroendocrine
D
GSE116918
L5_ssGSEA
L5_z-Score
BCRFS Probability
L5 High
L5 Low
p = 0.039
p = 0.005
Months
F
IHC staining
GS relative expression
(GS positive area)
***
Intact
Castration
WT
Pten-/-

(A) GLUL expression in patients pre- and post-ADT treatment. Data were retrieved from GSE48403. (B) UMAP plots showing various cell clusters in pre- and post-ADT PCa patients. Data were retrieved from GSE250189. (C) Violin plots showing KLK3 and GLUL expressions in all cell clusters. (D) Kaplan-Meier survival plots showing PCa patients with low and high scores of L5 gene signature. (E) Violin plots showing GLUL expression in all cell clusters. Data were retrieved from GSE227109. (F) Quantification of GS positive area in mouse prostate tissue IHC staining.

Fig. S3

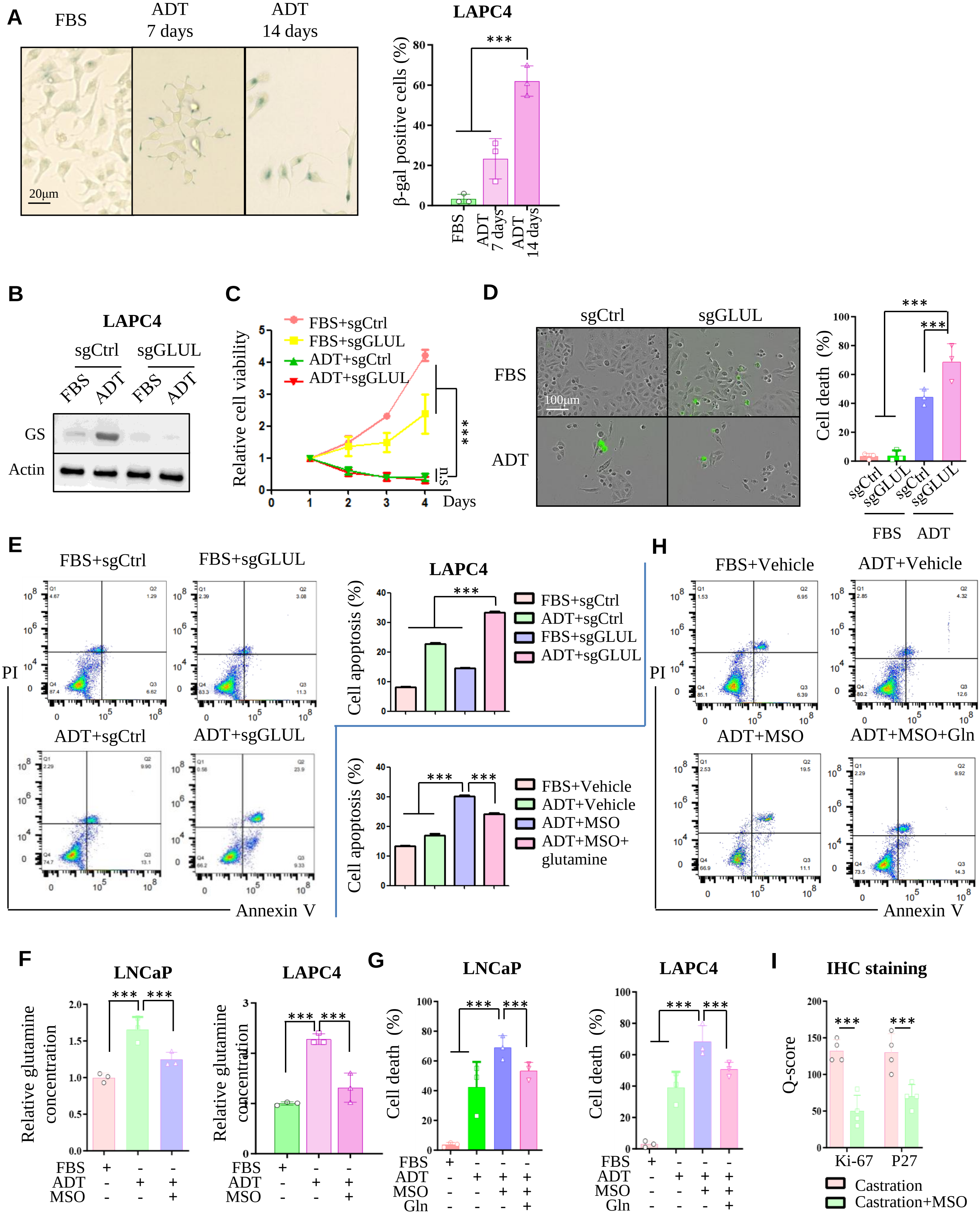

A
FBS
ADT 7 days
ADT 14 days
20μm
LAPC4
β-gal positive cells (%)
***
B
LAPC4
sgCtrl
sgGLUL
FBS
ADT
GS
Actin
C
FBS+sgCtrl
FBS+sgGLUL
ADT+sgCtrl
ADT+sgGLUL
Relative cell viability
Days
***
n.s
D
sgCtrl
sgGLUL
FBS
ADT
100μm
Cell death (%)
E
FBS+sgCtrl
FBS+sgGLUL
ADT+sgCtrl
ADT+sgGLUL
PI
Annexin V
LAPC4
Cell apoptosis (%)
FBS+Vehicle
ADT+Vehicle
ADT+MSO
ADT+MSO+ glutamine
H
FBS+Vehicle
ADT+Vehicle
ADT+MSO
ADT+MSO+Gln
F
LNCaP
LAPC4
Relative glutamine concentration
FBS
ADT
MSO
G
Cell death (%)
Gln
I
IHC staining
Q-score
Ki-67
P27
Castration
Castration+MSO

(A) β-staining showing representative images (left) and quantification (right) of positive cells. (B) Immunoblots showing loss of GS protein, achieved by CRISPR technique. (C) MTS assay showing cell viability in indicated treatment conditions. (D) Cytotox Green Dye staining showing dead cells which were positive for green dye. (E) Annexin V assay showing proportion of cell apoptosis in either ADT or GLUL-knockout alone or in combination. (F) Cellular glutamine concentration in LNCaP and LAPC4 cells treated with FBS, ADT or ADT+MSO. (G) The percentage of dead cells in indicated conditions. (H) Annexin V assay showing proportion of cell apoptosis in indicated conditions, achieved by flow cytometry. (I) Q-score quantification showing intensity of Ki-67 and P27 in mouse tissue. Data are depicted as mean ± s.d. * $P < 0.05$, ** $P < 0.01$ and ***$P < 0.001$.

Fig. S4

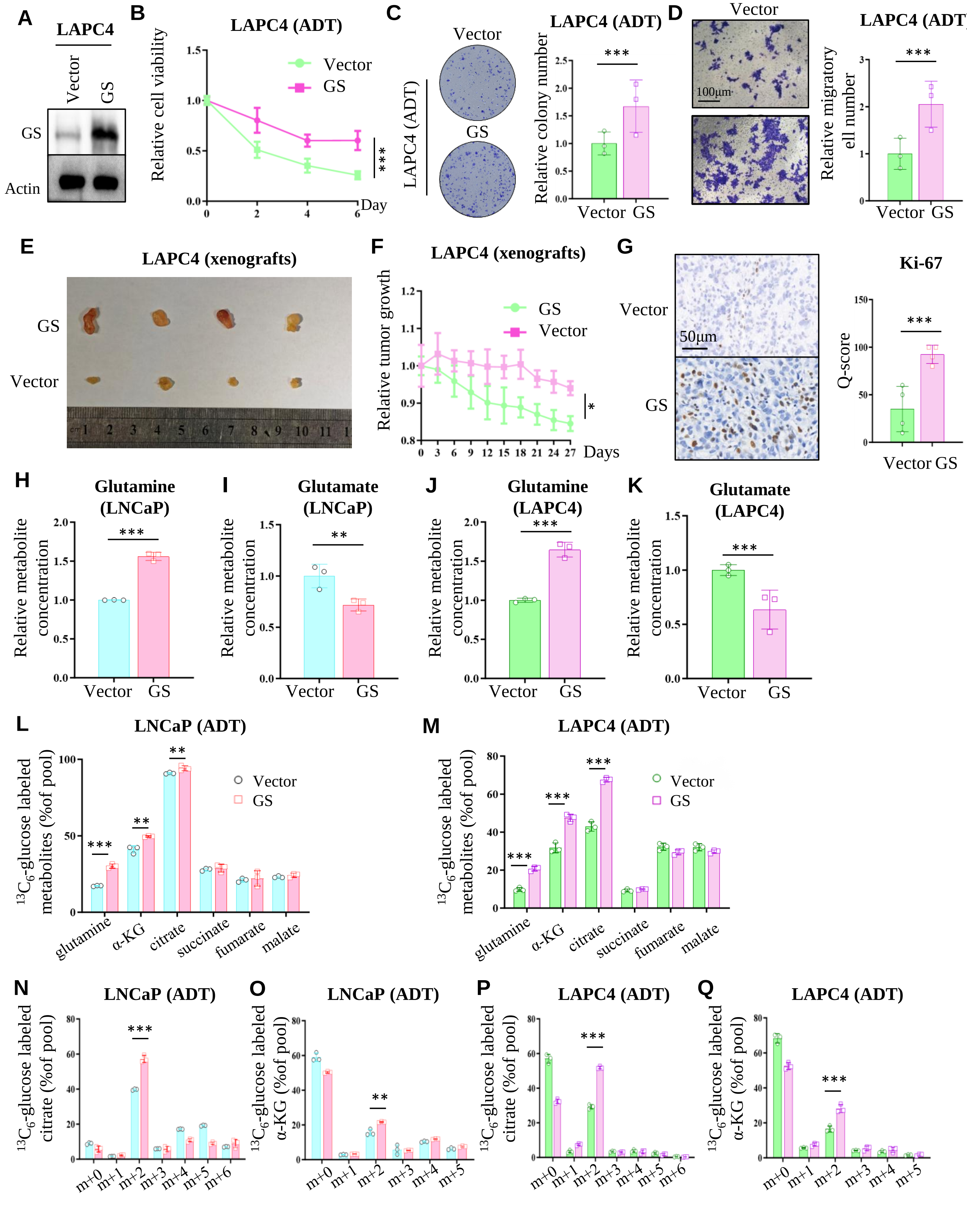
A
LAPC4
Vector
GS
GS
Actin
B
LAPC4 (ADT)
Relative cell viability
Vector
GS
***
Day
C
Vector
GS
LAPC4 (ADT)
LAPC4 (ADT)
Relative colony number
***
Vector
GS
D
Vector
100μm
LAPC4 (ADT)
Relative migratory ell number
***
Vector
GS
E
LAPC4 (xenografts)
GS
Vector
F
LAPC4 (xenografts)
Relative tumor growth
GS
Vector
*
Days
G
Vector
50μm
GS
Ki-67
Q-score
***
Vector
GS
H
Glutamine (LNCaP)
Relative metabolite concentration
***
Vector
GS
I
Glutamate (LNCaP)
Relative metabolite concentration
**
Vector
GS
J
Glutamine (LAPC4)
Relative metabolite concentration
***
Vector
GS
K
Glutamate (LAPC4)
Relative metabolite concentration
***
Vector
GS
L
LNCaP (ADT)
$^{13}C_6$-glucose labeled metabolites (%of pool)
Vector
GS
***
**
**
glutamine
α-KG
citrate
succinate
fumarate
malate
M
LAPC4 (ADT)
$^{13}C_6$-glucose labeled metabolites (%of pool)
Vector
GS
***
***
***
glutamine
α-KG
citrate
succinate
fumarate
malate
N
LNCaP (ADT)
$^{13}C_6$-glucose labeled citrate (%of pool)
***
m+0
m+1
m+2
m+3
m+4
m+5
m+6
O
LNCaP (ADT)
$^{13}C_6$-glucose labeled α-KG (%of pool)
**
m+0
m+1
m+2
m+3
m+4
m+5
P
LAPC4 (ADT)
$^{13}C_6$-glucose labeled citrate (%of pool)
***
m+0
m+1
m+2
m+3
m+4
m+5
m+6
Q
LAPC4 (ADT)
$^{13}C_6$-glucose labeled α-KG (%of pool)
***
m+0
m+1
m+2
m+3
m+4
m+5

(A-D) MTS assay, colony formation assay and cell migration assay showing cell viability, clonogenicity and migratory capability of GS overexpressed cells and vector cells in ADT conditions. (E-G) Representative images, relative tumor growth rate and tumor weights of GS overexpressed xenografts and vector xenografts after castration. (H) IHC staining showing Ki-67 expression in GS overexpressed xenografts and vector xenografts after castration. (I-L) Cellular glutamine and glutamate levels after GS was overexpressed. (M-N) $^{13}C_6$-glucose tracing analysis showing the incorporation of glucose into glutamine, α-KG, citrate, succinate, fumarate and malate in LNCaP and LAPC4 cells treated with ADT. (O-R) Fractions of $^{13}C_6$-glucose derived isotopologues in citrate andα-KG. Data are depicted as mean ± s.d. * $P < 0.05$, ** $P < 0.01$ and ***$P < 0.001$.

Fig. S5

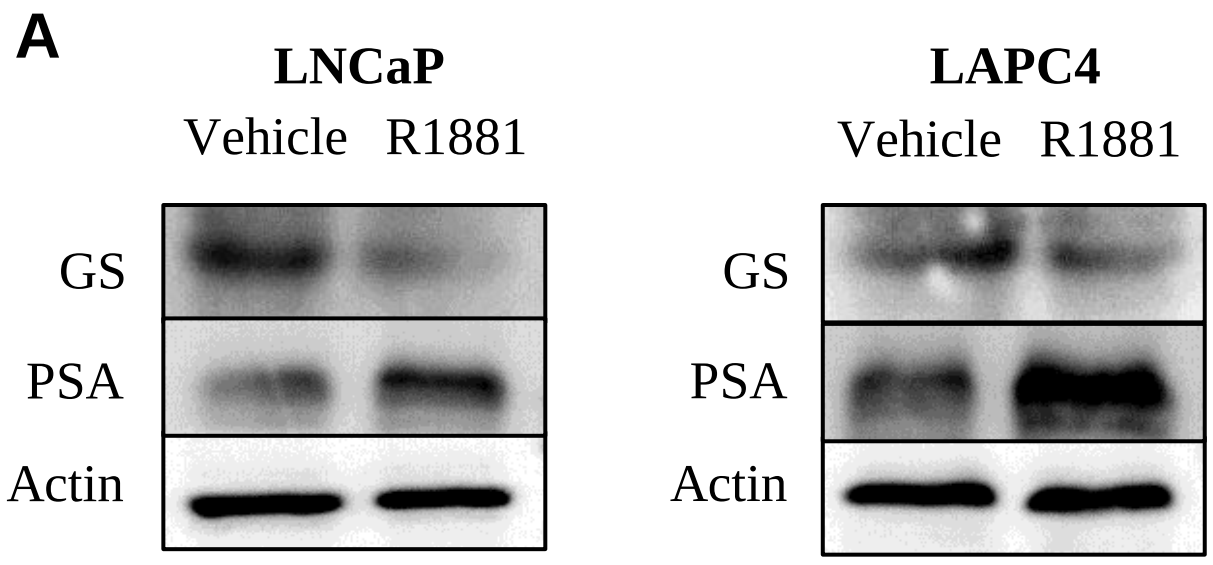

(A) Immunoblots showing GS protein expression in LNCaP and LAPC4 cells after R1881 (an AR stimulator) treatment. PSA expression was set as a positive control.
